# Effects of Corn Steep Liquor on the Fermentation Quality, Bacterial Community and Ruminal Degradation Rate of Corncob Silage

**DOI:** 10.3390/ani15233487

**Published:** 2025-12-03

**Authors:** Xinyi Wang, Xinfeng Wang, Tengyu Wang, Xiaoping Chen, Zuoxing Huang, Rui Yang, Shuai Liu, Xinwen Sun, Dengke Hua

**Affiliations:** 1 College of Animal Science and Technology, Shihezi University, Shihezi 832000, China; 15599814090@163.com (X.W.); wxf-4@163.com (X.W.); wangty142819@163.com (T.W.); 2 Tumushuke Tuxing Animal Husbandry Co., Ltd., Tumushuke 843806, China; 3 10th Division Livestock and Veterinary Workstation, Beitun 836000, China

**Keywords:** corn steep liquor, corncob, silage, bacterial community, ruminal degradation characteristics

## Abstract

This study aims to investigate the effect of varying addition levels of corn steep liquor (CSL) on the fermentation quality, bacterial community, and ruminal degradation rate of corncob silage. The experiment included a control group (CON) and four treatment groups: L1 with 5% CSL (50 g·kg^−1^ fresh matter), L2 with 10% CSL (100 g·kg^−1^ fresh matter), L3 with 15% CSL (150 g·kg^−1^ fresh matter), and L4 with 20% CSL (200 g·kg^−1^ fresh matter). The water content was controlled at 65% during fermentation for a period of 45 days. The results showed that the addition of CSL significantly increased the contents of dry matter (DM), crude protein (CP), and lactic acid (LA), while decreasing the pH, neutral detergent fiber (NDF), acid detergent fiber (ADF), and ammonia nitrogen (NH_3_-N). Furthermore, the addition of CSL altered the relative abundance of microbial genera. While *Pediococcus* was the dominant bacterium in the CON group, *Lactobacillus* became the prevalent species upon the addition of CSL, and its relative abundance increased in accordance with the supplemental amount. These findings suggest that CSL provides a favorable environment for lactic acid bacteria. It is worth noting that CSL addition did not significantly alter the phylum-level bacterial community structure. The dominant bacterial taxa across all treatments were *Bacillota*, *Proteobacteria*, and *Bacteroidota*, with their cumulative relative abundance accounting for over 95%. The rumen degradation of the tested feedstuff was determined using the in situ nylon bag method. Results revealed that incorporating CSL into corncob silage significantly enhanced the effective degradation rates of DM, CP, NDF, and ADF in the rumen of Kazakh sheep. Specifically, the effective degradation rate of DM in the CON group was only 49.10%, which increased to 53.12% following the addition of 20% CSL, along with corresponding improvements in the degradation rates of CP, NDF, and ADF. In summary, as a valuable feed additive, corn steep liquor supports the proliferation of beneficial microorganisms in fermentation systems by supplying essential growth substrates. Additionally, it improves the nutritional balance of corncob feed and further enhances the absorption and utilization of nutrients from this feed by animals.

## Simple Summary

China has experienced continuous expansion in the scale of livestock farming, accompanied by a corresponding surge in feed demand. This trend has intensified the competition for food resources between humans and livestock, making the development of unconventional feed sources an urgent priority. As the most widely cultivated grain globally, corn is known as the “king of grains”; As by-products of corn, corncobs and corn steep liquor represent significant resource potential. Corn steep liquor is rich in nutrients and can effectively enhance the nutritional quality of corncob silage. With increasing addition of corn steep liquor, the relative abundance of *Lactobacillus* in the silage increases accordingly, and the degradation rate of corncob silage in sheep rumen is significantly improved.

## 1. Introduction

Corn, an annual grass herb, is the most widely cultivated grain in the world. Its high yield, diverse sources, and low price make it the most processed crop with the longest industrial chain, earning it the title of the “king of grains”. Its applications span food production, animal feed, and various industrial uses [1]. In China, corn production has reached 260 million tons, with feed accounting for 71% of total corn demand. Corn by-products include corn steep liquor, corn husks, corn protein meal, and corn germ meal. The deep-processed products of corn are utilized in various industries, including food, textiles, automotive, electronics, and medicine [2]. As by-products of corn, corncobs and corn steep liquor represent significant resource potential. China generates 65 million tons of corncobs annually; however, most are discarded as agricultural waste, resulting in limited utilization [3], Corncobs are rich in crude protein, crude fiber, crude fat, minerals, and other nutrients, and they can serve as a potential source of roughage for animal feed [4].

Corncobs are the residual husks after corn is harvested, accounting for approximately 20% of the whole corn [5]. They consist of a hard outer shell and a complex, internal structure primarily composed of cellulose and hemicellulose. Corncobs exhibit strong water absorption, a uniform structure, good toughness, and high wear resistance, making them challenging to crush [6]. Due to their high fiber content and poor palatability as feed, livestock often encounter issues, such as selective feeding and leftover feed, which greatly limits the utilization of corncobs as roughage [7]. Studies have indicated that fermentation of corncobs leads to a looser structure, with more porous surfaces and rough textures, suggesting that the breakdown of cellulose, hemicellulose, and lignin facilitates better microbial utilization and potentially improves their palatability [8]. Microbial fermentation can balance the nutritional structure of feed, enhance palatability, increase feed efficiency, and reduce the prevalence of intestinal pathogens. The fermentation process softens the texture of corncob and enhances feed palatability [9]. Research findings have demonstrated that, compared with the conventional silage group, dairy cows supplemented with corncob and corn husk silage exhibited a 2.42% increase in milk production, indicating that the nutritional value of corncob and corn husk silage surpasses that of traditional corn stover silage [10]. As an unconventional and low-cost feed resource, corncob can effectively replace corn silage, alleviating shortages of traditional feed materials such as corn and soybean meal [11]. However, corncobs have low crude protein, and their fibers are not easily utilized. Adding corn steep liquor can enhance the nutritional components of the feed and make better use of resources.

Corn steep liquor (CSL) is a by-product of wet processing of corn starch, produced by soaking corn in a sulfite solution [12]. This by-product CSL is rich in soluble protein, sugar, vitamins, minerals, and other nutrients [13], making it a valuable and cost-effective nutrient source for antibiotic fermentation and MSG production [14]. CSL can be produced at a low cost, it has been proposed as a nitrogen source for the biochemical industry to replace more expensive nitrogen sources. It is a mixture of corn extracts that are steeped in water, with the soluble solids varying from 3% to 12% (dry basis), and it is a good source of sugar 2.5% (dry basis), organic acids 26% (dry basis), crude protein 47% (dry basis), minerals, and vitamins, which makes it highly beneficial as a source of nutrients for microorganisms [15]. Studies show that supplementing urea-treated wheat straw with CSL improves the digestibility of dry matter, organic matter, crude fiber, and crude protein in buffalo [16]. Experiments were conducted with mixed CSL with corn straw for silage at varying CSL concentrations of 0%, 15%, 20%, 25%, and 30%. The results showed that a CSL concentration of 15–20% yielded the best fermentation quality with higher protein levels in the straw, while higher CSL proportions inhibited lactic acid bacteria growth and impaired feed fermentation [17]. Research has shown that CSL could effectively replace 75% of fishmeal without negatively impacting fish growth. Dietary CSL supplementation improved the protein and fat content at slaughter and elevated nutritional quality [18]. The addition of CSL to fermented apple residue can improve the nutritional conditions for microbial growth in the fermentation substrate. This promotes the proliferation of microorganisms, stimulates the development of enzyme-producing fungi, and increases the synthesis and secretion of extracellular enzymes. As a result, it facilitates the hydrolysis of macromolecular carbohydrates in apple residue and boosts the production of single-cell proteins [19]. CSL is rich in vitamins, serving as an effective growth promoter for microorganisms. Through fermentation, it increases the crude protein content in feed, supports microbial growth, and provides a nitrogen source for feed supplementation [20]. However, little information is available relating to the microbial community and ruminal degradation characteristics during the ensiling of corncob. With the rapid extension of cattle and sheep farming and the rising cost of forage, feed expenses have surged. The rational development and utilization of unconventional feed sources, such as agricultural and forestry by-products, can diversify forage options, alleviate the shortage of conventional feed materials, reduce feed costs, and generate significant economic value [21].

In this study, corncobs were used as the feed substrate for ensiling, supplemented with varying proportions of CSL and microbial inoculants. The objective was to investigate the effects of varying CSL addition levels on the silage quality and microbial community of corncob. By assessing these variations’ impact on silage quality and microbial flora, this research strives to convert agricultural waste, such as corn steep liquor and corncobs, into usable feed, thereby enhancing the efficiency of corn resource utilization. Ultimately, it aims to provide a theoretical foundation for the feed utilization of corn steep liquor and corncobs.

## 2. Materials and Methods

### 2.1. Ethics Statement

All animals involved in this experiment were cared for in accordance with the guidelines of the Biology Ethics Committee of Shihezi University, Shihezi, Xinjiang, China. The experimental procedures were reviewed and approved by the committee (A2025-713).

### 2.2. Materials Preparation

Corncobs were collected from Bole Zonghai Jiahui Feed Company in May 2024 and crushed to a size of 1–3 cm. Corn steep liquor was purchased from Xinjiang Wujiaqu Meihua Biological Company; its production batch number was YL01-241005. All these raw materials were then transported to the experimental station for fermentation experiments. The nutritional composition of raw materials is shown in Table 1.

### 2.3. Fermentation Process

A single-factor experimental design was employed in this study. The control group (CON) was corncob silage without additive supplementation, while L1, L2, L3, and L4 represented experimental groups, with corn steep liquor in varying concentration gradients (L1: 5%, L2: 10%, L3: 15%, L4: 20%, based on total weight) added into the corn silage. For each treatment group, 0.5% microbial preparations (including *Saccharomyces cerevisiae* at an application rate of 2.5 × 10^8^ CFU/mL, *Bacillus subtilis* at an application rate of 5.5 × 10^8^ CFU/mL, *Lactobacillus plantarum* at an application rate of 2.5 × 10^8^ CFU/mL, *Geotrichum candidum* at an application rate of 7.5 × 10^8^ CFU/mL, and *Candida utilis* at an application rate of 1.0 × 10^8^ CFU/mL) were added as inoculum. All strains were purchased from the Institute of Microbiology, Chinese Academy of Sciences (CAS) and along with 0.2% salt (based on total weight) were added as energy source for microbes. The samples were evenly mixed until the moisture content reached 65%, with a total weight of 1000 g per group, and five repetitions were conducted. After thorough mixing, samples were placed into polyethylene vacuum bags, sealed with a vacuum packaging machine, and fermented under dark conditions at room temperature for 45 days.

### 2.4. Chemical Composition Analysis

After 45 days of fermentation, 200 g samples from each group were dried in the oven at 65 °C for 48 h to determine the dry matter (DM) content. Crude protein was determined using an N310 automatic Kjeldahl nitrogen analyzer, and was calculated by multiplying TN by 6.25 [22]. Soluble sugars were determined using the anthrone–sulfuric acid method, while neutral detergent fiber (NDF) and acid detergent fiber (ADF) contents were assessed according to the Van Soest method [23]. Extract content was detected via the Soxhlet method, and crude ash was determined based on the GB/T 23742-2009 standard [24].

### 2.5. Fermentation Quality Analysis

After 45 days of silage, 20 g samples were taken from each treatment and mixed with 180 mL of distilled water. The mixture was stored in a refrigerator at 4 °C for 24 h and then filtered through four layers of gauze and qualitative filter paper. The resulting filtrate was defined as the feed extract. The pH value of this extract was measured using a pH meter (PHBJ- 260F; Shanghai INESA Scientific Instrument Co., Ltd., Shanghai, China), while the ammonia nitrogen (NH_3_-N) content was analyzed using colorimetry, specifically, the phenol–hypochlorite method [25]. Volatile acids were determined using a high-efficiency gas chromatograph (Agilent Technologies 7890A, Santa Clara, CA, USA), and lactic acid content was assessed using a lactic acid kit from Nanjing Jiancheng Bioengineering Institute.

### 2.6. Microbial Community Analysis

For this procedure, 20 g of silage corncob was placed in a conical flask with 180 mL of ultra-pure water, soaked in a shaker at 4 °C for 4 h, then filtered through four layers of gauze and qualitative filter paper. The mixture was centrifuged at 10,000 rpm (≈12,800× *g*) for 15 min at 4 °C for DNA extraction and collect the supernatant solution. Microbial DNA was isolated according to the Fast DNA ^®^Spin Kit for Soil (Shanghai BIOZERON Co, Ltd., Shanghai, China), with quality assessed through agarose gel electrophoresis. DNA concentration and purity were determined using a Nano Drop 2000. PCR amplification of the V3–V4 regions of bacterial 16S rRNA genes was accomplished using primers 341F (5’- CCTAYGGGRBGCASCAG-3’) and 806R (5’- GGACTACNNGGGTATCTAAT-3’). The PCR products were quantified using a Quanti Fluor™ ST Blue fluorescence quantification system (Promega, Madison, WI, USA) and mixed according to the sequencing volume requirements. Finally, a PE250 library was constructed, and sequencing was conducted by Shanghai Ling En Biotechnology Co. Ltd (Shanghai, China). The original sequencing reads have been archived in the NCBI Sequence Read File (SRA) database. Sequencing was performed on the Illumina PE250 platform (Shanghai BIOZERON Co, Ltd., Shanghai, China). The obtained data were analyzed using QIIME2 software. The generated sequence data are available in the Sequence Read File (SRA) under the accession number PRJNA1269722.

### 2.7. Ruminal Degradation Analysis

Three rumen-cannulated Kazakh sheep (12-month-old, 25 ± 5 kg) were used in the in situ trial to determine the digestibility of the nutrients. The animals were raised in the experimental animal station of Shihezi University and underwent quarantine and deworming before the trial. Animals were fed twice daily at scheduled times (9:00 and 18:00), with free access to water. The basal diet was formulated according to the nutritional requirements specified in NY/T816-2021, maintaining a concentrate-to-roughage ratio of 4:6 [26]. Dietary composition is shown in Table 2.

The ruminal degradation rate was determined using the in vivo method, also referred to as the in situ nylon bag technique. For this purpose, 3 g of air-dried samples were placed into labeled nylon bags (12 × 8 cm, 40–50 µm pore size), which were subsequently sewn shut. Each sample was repeated three times. Each set of three parallel samples was attached to one end of a plastic hose, with the other end secured to a thick nylon rope, which was then fixed on the iron ring at the outer end of the rumen fistula to prevent slipping. After being placed in the rumen for 4, 8, 12, 24, 36, and 48 h, feeds were removed and washed under cold water for 10-15 min until clear. At the zero-hour mark, the sample were placed in a 37 °C water bath, stirred slowly every 5 min, and removed after 30 min. The samples were rinsed slowly under running water until clear, and the rinsed nylon bags were placed in an oven to dry for 24 h before weighing. Parameters such as a, b, c, and the effective degradation rate (ED) of DM, NDF, ADF, and CP were determined. The ruminal disappearance rate was calculated as follows:

$$A\;=\;\frac{B-C}{B}\;\times \;100\%$$

where A represents the nutrient disappearance rate (%), B represents the initial nutrient content (g), and C represents the post-degradation nutrient content (g). The rumen degradation rate was calculated via the following equation:

y = a + b × (1 − e^−c t^)

where y represents the degradation rate after t hours, a represents the rapidly degraded portion, b represents the slow degradation portion, c represents the degradation velocity constant of the slow degradation portion. The effective degradation rate of the sample was calculated as follows:

ED = a + b × c/(c + k)

where ED is the effective degradation rate in the rumen and k is the feed outflow rate of 0.0253/h in this experiment [27].

### 2.8. Statistical Analysis

In this experiment, SPSS (version SPSS 27.0, Chicago, IL, USA) was used for univariate analysis of variance to examine the effects of varying proportions of corn steep liquor on fermentation quality and microflora in corncobs. The data of the rumen degradation rate were analyzed by one-way ANOVA and a nonlinear model. The correlation diagram of microbial communities was created using Origin 2021 (OriginLab Corporation, Northampton, MA, USA), while multiple comparisons were conducted through Duncan’s method. A significance level of *p* < 0.05 was established to denote significant differences, and *p* < 0.01 indicates an extremely significant difference. Results are presented as means and standard error of the mean (SEM).

## 3. Results and Discussion

### 3.1. Nutrient Composition of the Ensiled Mixture

As shown in Table 3, corn steep liquor had a significant impact on all indices of corncob silage. With increasing CSL supplementation, the DM, CP, and ash contents significantly increased (*p* < 0.05), while the contents of WSC, EE, NDF and ADF significantly decreased (*p* < 0.05). For optimal feed fermentation, moisture content should be about 65%; excessive moisture can lead to mold deterioration, while high DM content can effectively inhibit the growth of harmful microorganisms [28]. In this study, the highest DM percentage was observed in the L3 group at 36.84%, significantly surpassing that of other groups, with both L2 and L3 groups showing improved DM levels compared to the CON group (*p* < 0.05). This effect can be attributed to the DM content present in the CSL, which, along with higher CSL additions, promoted fermentation and reduced DM consumption by microorganisms. This aligns with the finding from [29], where the DM content in the corncob treated group was significantly higher than that in control group, due to the rapid proliferation of beneficial microorganisms such as lactic acid bacteria.

CP is a critical indicator of nutritional value and reflects nutrient retention and loss. The CP content increased with higher CSL supplementation, peaking at 7.83% in the L4 group, significantly higher than that of other groups (*p* < 0.05). CP content in experimental groups was significantly higher than that in CON group (*p* < 0.05). This is consistent with findings from [30], where CSL was shown to enhance nitrogen fixation and significantly increase CP content in corn stalks. Aiming to achieve a WSC content of at least 5% dry matter for proper fermentation [31], we noted that a significant decrease in WSC contents increased the CSL, with the lowest WSC content of 2.77% observed in the L2 group, significantly lower than that in other groups (*p* < 0.05). In this experiment, the WSC content in the treatment group was significantly lower than that in the control group. This was because as the corn pulp was added, the microorganisms grew rapidly and consumed the WSC content in the feed very quickly. As the addition ratio of CSL increased, the total amount of minerals introduced rose accordingly, thereby leading to an upward trend in the crude ash content of the mixed silage materials.

The content of coarse fiber serves as an indicator of roughage utilization difficulty, with NDF and ADF levels being negatively related to feed quality [32]. As CSL addition increased, NDF and ADF contents decreased significantly, achieving 62.79% and 30.41% in the L4 group, compared to 74.99% and 37.23% in the CON group, respectively (*p* < 0.05). CSL promotes the growth of cellulose-decomposing bacteria such as *Saccharomyces cerevissae*, leading to structural loosening in fiber after 45 days of fermentation, which accelerates NDF and ADF degradation [33]. Overall, CSL significantly reduced the NDF and ADF contents, optimizing the nutritional quality of the corncob.

### 3.2. Fermentation Quality of the Ensiled Mixture

It can be seen from Table 4 that after 45 days of fermentation, the pH significantly decreased with increased CSL levels, with test groups exhibiting lower pH compared to the CON group (*p* < 0.05). The pH values of the L2, L3, and L4 groups did not differ significantly, with the L4 group’s lowest pH of 4.09 comparable to the CON group’s pH of 4.48. pH is an important indicator of feed quality, with optimal silage typically exhibiting pH values between 3.8 and 4.2. A lower pH inhibits the growth of harmful microorganisms [34]. However, the pH value of silage depends on several characteristics, such as the content of buffering substances (from crops or produced during fermentation) and other organic acids, the application of additives, and moisture content. Drier silage usually has a higher pH value under good fermentation conditions. Whole-plant corn silage typically has a lower pH value due to its higher carbohydrate content and more sufficient lactic acid bacteria fermentation [35]. In this study, the control group without CSL addition exhibited a higher pH value than the CSL supplemented groups. As an acidic substance, CSL contributed to the rapid decrease in pH with higher CSL addition, creating favorable conditions for beneficial microorganisms like lactic acid bacteria. Previous studies have reported that increased CSL proportions led to reduced pH levels in corn stalks [17].

It has been reported that a higher ratio of ammonia nitrogen (NH_3_-N) leads to higher protein decomposition and worse feed quality [36]. The NH_3_-N ratio in the feed decreased gradually with increasing CSL levels, with the L3 and L4 groups displaying significantly lower ratios compared to the other groups (*p* < 0.05). In this study, the L4 group exhibited the lowest NH_3_-N ratio, indicating minimal protein decomposition, whereas the CON group demonstrated the highest ratio, suggesting the most significant protein breakdown rates, with no significant differences observed among other groups. It has been found that an acidic environment inhibits protein hydrolysis and reduces the concentration of NH_3_-N [31]. In this experiment, the supplemented CSL contributed to the lower pH value, thereby reducing protein hydrolysis.

LA content significantly increased with higher CSL ratios (*p* < 0.05), peaking at 4.87 in the L4 group, with all treatment groups exceeding the CON group’s LA content of 2.05. Although no significant differences were observed among groups regarding AA content (*p* > 0.05), the L3 group had the highest AA content, while the L1 group had the lowest. The content of PA in the L2 group was the highest and significantly higher than that in the L4 group (*p* < 0.05). No BA was detected in any group. AA plays an important role in silage aerobic stability; higher concentrations of LA and AA are preferred, as lower BA content enhances silage quality [32]. In this experiment, variations in AA content were not significant across groups, while lower PA content also contributed positively to silage stability [37]. The LA/AA ratio in Group L4 was significantly higher than that in the other groups. A desirable LA/AA ratio exceeding 3:1 is typically indicative of a more dominant homolactic fermentation process. In the present study, the LA/AA ratios of all groups were less than 3, suggesting that heterofermentative lactic acid bacteria predominated in the fermentation system, which contributed to the relatively high aerobic stability of the silage. This finding is consistent with the results reported by Li et al. [38]. The results indicated that there was no statistically significant difference in AA/PA levels between the experimental groups and the control group in this study.

### 3.3. Microbial Communities of the Ensiled Mixture

**Figure 1:**
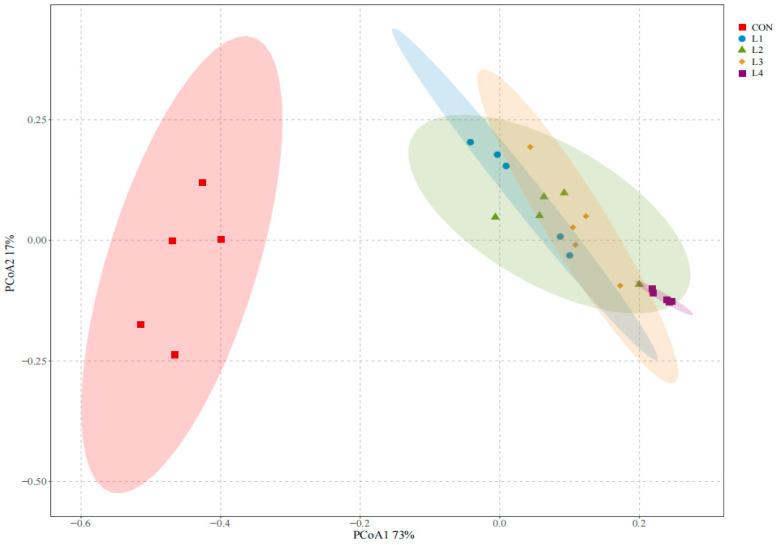
**PCOA of microbial communities in corncob silage supplemented with various levels of corn steep liquor.** Note: CON represents the control group; L1 denotes the group with 5% CSL added; L2, L3, and L4 correspond to the groups supplemented with 10%, 15%, and 20% CSL. Different colors or shapes indicate different groupings.

A total of 2726 OTUs were identified in this study. As shown in Table 5, groups treated with L4 exhibited significantly higher Chao1 and ACE indices than the CON group (*p* < 0.05), while no significant differences were observed among other groups, with the lowest Chao1 and ACE values in the CON group and the highest in L4. The Shannon value was the highest in the CON group; however, groups L2, L3, and L4 exhibited significantly lower Shannon indices (*p* < 0.05), indicating reduced microbial diversity in treatment groups with higher CSL levels. The Simpson index in the L4 group was significantly higher than that in the other groups, while the CON group recorded the lowest Simpson index of 0.12. The Chao 1 index indicates microbial richness; the higher the Shannon index, the richer the community diversity; the lower the Simpson index, the richer the community diversity; the higher the ACE index, the greater the number of microorganisms [39]. The addition of CSL enriched the bacterial community in the feed but led to a reduction in microbial diversity. In this study, coverage rates of all groups exceeded 99%. Additionally, the β-diversity exhibited significant differences between the CON group and other treatments, as shown in Figure 1 (*p* < 0.05).

### 3.4. Bacterial Community in the Ensiled Mixture

**Figure 2:**
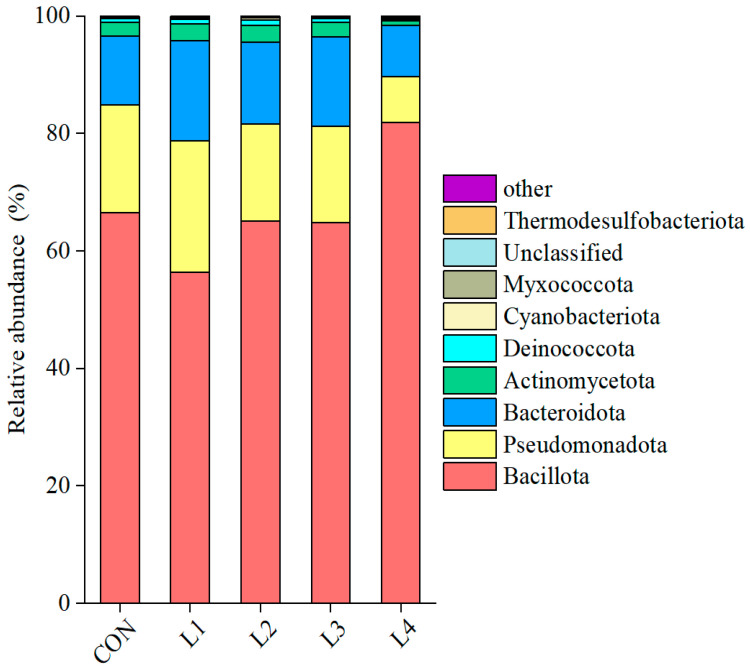
**Relative abundance of bacterial phyla in corncob silage supplemented with various levels of corn steep liquor**. Note: CON represents the control group; L1 denotes the group with 5% CSL added; L2, L3, and L4 correspond to the groups supplemented with 10%, 15%, and 20% CSL.

The relative abundance of bacteria in corncob silage across different treatments is shown in Figure 2. At the phylum level, the top three bacteria in all treatments were *Bacillota*, *Proteobacteria*, and *Bacteroidota*, collectively exceeding 95% relative abundance. CSL addition did not significantly alter the bacterial community structure at the phylum level, and bacterial abundance exhibited minimal variation between the CON, L2, and L3 groups. However, the L1 group contained the lowest abundance of *Bacillota* but higher levels of *Proteobacteria* and *Bacteroidota*, accounting for 22% and 17%, respectively. Conversely, the L4 group exhibited the highest relative abundance of *Bacillota* at 82%, while the relative abundance of *Proteobacteria* and *Bacteroidota* dropped to 8% and 9%. Notably, adding 20% CSL increased *Bacillota* levels, whereas the addition of 5% CSL resulted in reduced relative abundance of *Bacillota* and increased relative abundance of *Proteobacteria.*

**Figure 3:**
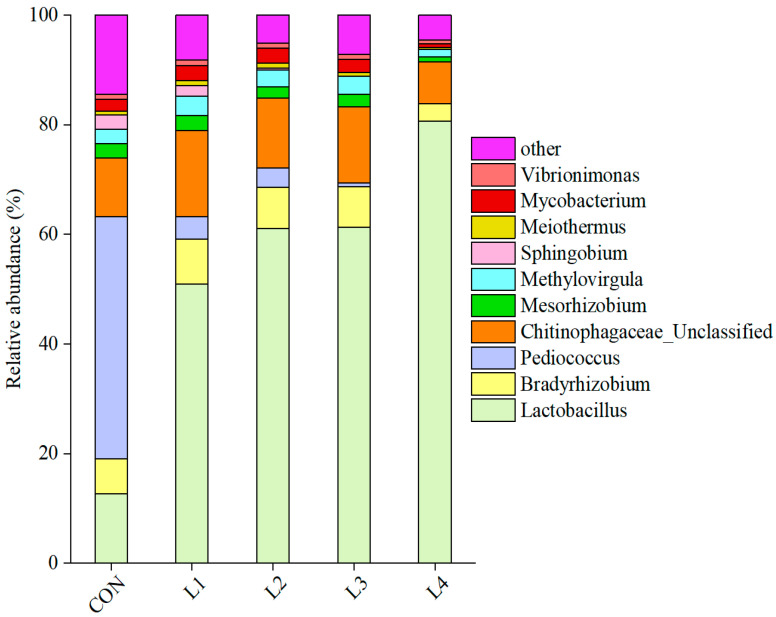
**Relative abundance of bacterial genera in corncob silage supplemented with various levels of corn steep liquor**. Note: CON represents the control group; L1 denotes the group with 5% CSL added; L2, L3, and L4 correspond to the groups supplemented with 10%, 15%, and 20% CSL.

The relative abundance of bacterial communities at the genus level is shown in Figure 3. In the CON group, the dominant bacterial genera were *Pediococcus*, *Lactobacillus*, and *Chitinophagaceae_Unclassified.* In contrast, the three dominant genera in the CSL treatment groups included *Lactobacillus*, *Chitinophagaceae_Unclassified*, and *Bradyrhizobium*. The relative abundance of *Lactobacillus* was only 13% in the CON group, significantly lower than that in other treatment groups, where *Pediococcus* reached 44%. The shift in dominant bacteria from *Pediococcus* to *Lactobacillus* upon CSL addition is noteworthy. The L4 group’s relative abundance of *Lactobacillus* was 81%, with other treatment groups ranging between 50–65%, all significantly higher than that observed in the CON group. The fermentation process is primarily driven by microorganisms, with numerous microbial species participating to form a complex symbiotic system [29]. Consequently, CSL supplementation altered the fermentation process by enhancing *Lactobacillus*, which is crucial for LA production and pH reduction. *Pediococcus*, a gram-positive bacterium capable of growing in both aerobic and anaerobic environments, may help regulate the intestinal bacterial compositions and improve the overall immune function. Furthermore, CSL addition can inhibit the growth of *Pediococcus* in diets [40].

### 3.5. Correlation Analysis of Differential Bacteria and Fermentation Parameters

**Figure 4:**
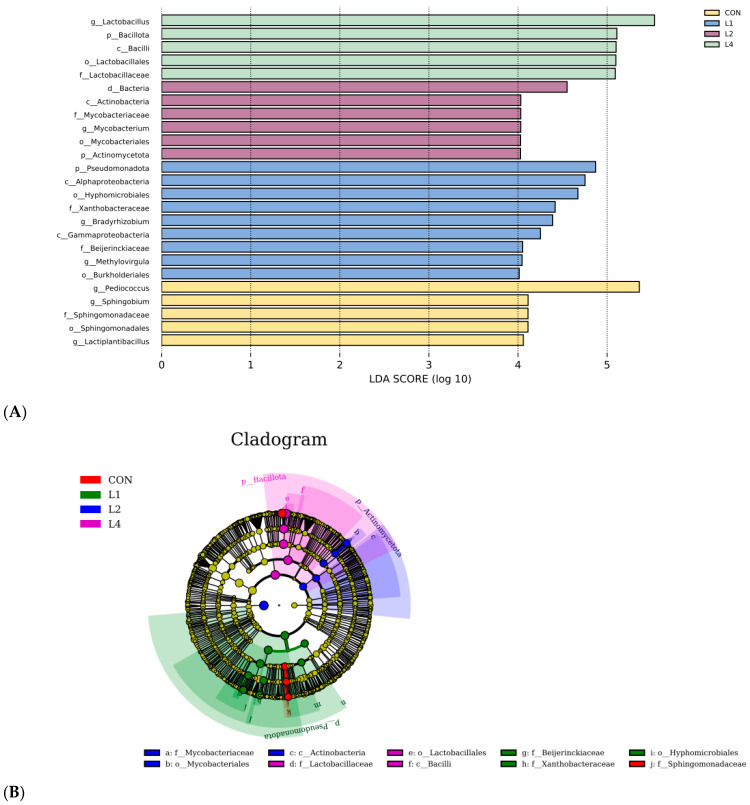
LDA histogram and evolutionary branching diagram of microbes in corncob silage supplemented with various levels of corn steep liquor. Note: (**A**) represents the distribution of LDA values of different treatments, and the LDA discrimination histogram counts the categories of microorganisms with significant effects in each group. The longer the histogram, the greater the difference effect of species abundance. Yellow represents the CON group, blue represents the L1 group, purple represents the L2 group, and green represents the L4 group. (**B**) The dots radiating from the inner circle to the outer circle represent the taxonomic level from phylum to genus. Each dot at different taxonomic levels represents a taxonomic group at each level. The diameter of the circle reflects the relative abundance of the taxonomic group, and the differences are not significant. The blue dots represent the microbiomes that play an important role in the L2 group, the green dots represent the microbiomes that play an important role in the L1 group, and the purple dots represent the microbiomes that play an important role in the L4 group.

**Figure 5:**
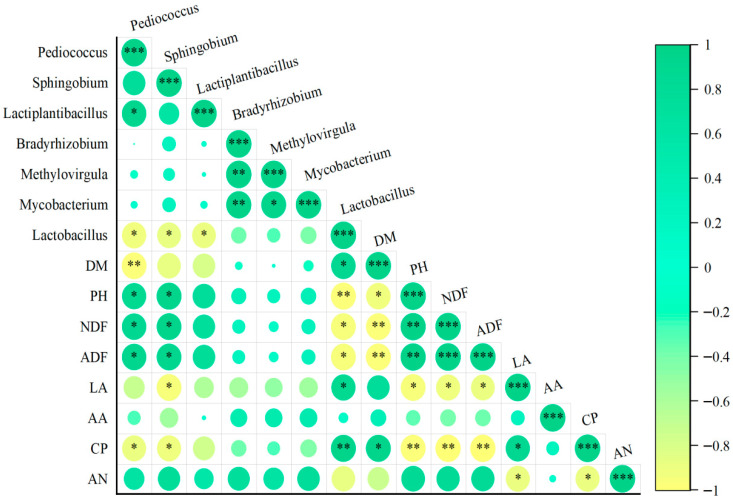
Spearman correlation heat map between bacterial genera and fermentation indexes in corncob silage supplemented with various levels of corn steep liquor. Note: Different colors represent the degree of correlation, green represents the positive correlation, yellow represents the negative correlation; * *p* < 0.05, ** *p* < 0.01, and *** *p* < 0.001.

LEfSe analysis was used to evaluate the differential microorganisms in each treatment group, identifying species with an LDA value greater than 4 and a *p*-value less than 0.05 as differential species, as shown in Figure 4. A total of seven differential genera emerged, with the CON group containing genera such as *Pediococcus*, *Sphingobium*, and *Lactiplantibacillus*. The L1 group presented *Bradyrhizobiu* and *Methylovirgula*, while the L2 group included *Mycobacterium*. The distinct genus identified in the L4 group was *Lactobacillus*, with no differences noted in the L3 group. The addition of CSL significantly altered the microbial abundance across the groups. A Spearman correlation heatmap (Figure 5) illustrates the relationships between the seven genera and eight fermentation parameters, with green representing positive correlations and yellow indicating negative correlations. *Pediococcus* positively correlated with pH, NDF, and ADF but negatively correlated with CP and DM, while *Sphingobium* exhibited similar positive correlations but negatively correlated with LA and CP. *Lactobacillus* was positively correlated with DM, LA, and CP but negatively correlated with pH, NDF, and ADF. *Lactobacillus* species, including *Levilactobacillus*, *Loigolactobacillus*, *Pediococcus*, *Lacti-plantibacillus*, *Lentilactobacillus*, and *Latilactobacillus*, are known for their ability to rapidly decrease pH [41]. Adding CSL provided a suitable growth environment for lactic acid bacteria.

### 3.6. Ruminal Dry Matter Degradation Characteristics of Corncob Silage

The ruminal degradation rate is an important indicator to evaluate the nutritional value of ruminant feed. Higher degradation rates signify improved digestibility, facilitating nutrient accessibility [42]. As shown in Table 6, increasing CSL supplementation correlated with elevated dry matter degradation rates, surpassing the CON group in all treatment groups. The L4 group exhibited the highest dry matter degradation rate at 12 h, significantly exceeding those of other groups (*p* < 0.05). Additionally, the rapid degradation rates for DM in the L3 and L4 groups were significantly higher than that in other groups (*p* < 0.05), recording values of 7.23 and 6.78, respectively. The L4 group achieved a maximum effective degradation rate of 53.12%, significantly higher than that in other groups (*p* < 0.05). Compared to the CON group, the effective DM degradation rates for the L3 and L4 groups increased by 2.28% and 8.19%, respectively. These results underscore the positive impact of CSL supplementation on feed digestibility and utilization by rumen microorganisms. While the high fiber content of corncobs traditionally limits their effectiveness as roughage, the inclusion of CSL reduces NDF and ADF contents, enhancing their nutritional value. Combing these agricultural by-products into silage offers notable nutritional benefits, with treatment groups exhibiting significantly higher DM degradation compared to the CON group [43]. The L4 group recorded the highest DM degradation rates during the initial 4 to 12 h ago, attributed to lower fiber content, which facilitated absorption by microorganisms. In contrast, the CON group exhibited significantly lower rapid and slow degradation rates, indicating a higher presence of undecomposable substances and a reduced effective degradation rate.

### 3.7. Ruminal Crude Protein Degradation Characteristics of Corncob Silage

As shown in Table 7, the CP degradation rates of each treatment group significantly exceeded those of the control group at all time points, with degradation rates for all treatment groups at 48 h ranging from 71% to 75%, mirroring trends observed in DM degradation. The highest degradation rate in the L4 group was noted at 24 h and 36 h, remaining elevated at 48 h (74.69%). The L1 group exhibited the highest rapid and slow degradation rates, significantly surpassing other treatment groups (*p* < 0.05). The effective degradation rates for the L2, L3, and L4 groups exceeded 52%, significantly higher than those of the CON and L1 groups (*p* < 0.05). Microbial degradation of protein and synthesis of microbial protein are vital sources of animal protein, influenced by various factors within the complex ruminal ecosystem. Feed proteins comprise both rumen degradable and undegradable portions; rumen-degradable proteins can be broken down into smaller peptides, amino acids, and ammonia nitrogen by rumen microorganisms, whereas undegradable proteins cannot be effectively utilized [44]. Enhanced CP degradation rates in treatment groups suggest an increased availability of rumen degradable proteins in CSL that can be efficiently absorbed and utilized by rumen microorganisms. The consistently lower CP degradation rate in the CON group indicates a higher proportion of undegradable proteins in the corncob silage, resulting in a lower effective degradation rate.

### 3.8. Ruminal ADF and NDF Degradation Characteristics of Corncob Silage

ADF and NDF contents primarily originate from cell wall structures, and their degradation rates in the rumen reflect the feed’s digestibility [45]. As shown in Table 8, the degradation rates of the CON group at 4 h and 8 h were significantly lower than those of the treatment groups (*p* < 0.05), while the L4 group exhibited the highest. After 48 h, the NDF degradation rate in the L4 group peaked at 62.85%, compared to approximately 55% in other groups. Additionally, the rapid and slow degradation rates in the L3 group were significantly higher than those in other groups (*p* < 0.05), indicating the highest effective degradation rates of 40.76% and 43.25% for the L3 and L4 groups, respectively, both significantly exceeding the CON group values, by 12.19% and 19.05%. As shown in Table 9, the addition of CSL markedly increased ADF degradation rates across all treatment groups compared to the CON group at various time points, with L4 group consistently outperforming other groups, particularly at 8, 12, and 48 h (*p* < 0.05). The rapid degradation rate and effective degradation rates were the highest in the L3 and L4 groups, while the CON group displayed the lowest effective degradation rate; effective degradation rates in the CSL treatment groups increased by 5.97% to 16.34%. These observations suggest that the lower ADF and NDF degradation rates in the CON group resulted from higher fiber content, which inhibits microbial decomposition. Generally, the NDF degradation rates exceeded those of ADF, consistent with common digestive traits. Notably, the ADF and NDF degradation rates approached each other at 36 h, indicating that substances with digestible potential were degraded within the first 4 to 12 h, with subsequent stabilization in degradation rates after 48 h. The addition of CSL significantly enhanced the degradation rates of DM, CP, NDF, and ADF in the rumen. Previous research [44] revealed that lower NDF content in feed was associated with a higher degradation rate of NDF at 48 h, and the degradation rate of NDF in the feed was higher than that of ADF. The main components of NDF are cellulose, hemicellulose, and lignin; hemicellulose is the main fermentable component, and lignin cannot be utilized by rumen microorganisms. ADF in feed is mainly composed of lignin, cellulose, and silicon dioxide. Lignin, due to its unique structure, cannot easily be decomposed by rumen microorganisms [46].

## 4. Conclusions

This study demonstrated that supplementing 20% CSL was the most effective proportion to significantly increase DM, CP, and LA values while significantly lowered pH, NH_3_-N ratio, NDF, and ADF levels in corncob silage. Additionally, CSL significantly altered the microbial community at the genus level, with the L4 group showing the highest relative abundance of *Lactobacillus*. Overall, the ruminal degradation rates of DM, CP, NDF, and ADF improved with the addition of CSL, highlighting its potential to enhance the nutritional value of silage and improve feed degradation rate. A limitation of this study is that the maximum addition level of CSL was set at 20%, and the effects of higher CSL concentrations (25–30%) on corncob silage were not investigated. Furthermore, the scientific value of this work could have been further enhanced by complementary feeding experiments to assess the silage’s effects on rumen microbial communities and animal productivity.

## Figures and Tables

**Table 1:** Nutritional components of raw corncob and corn steep liquor (%DM).

Items	Corncob DM%	Corn Steep Liquor
DM (%)	93.21	62.58
CP (%)	3.73	24.03
NDF (%)	74.45	—
ADF (%)	38.91	—
WSC (%)	3.64	4.62

Note: DM, dry matter; CP, crude protein; NDF, neutral detergent fiber; ADF, acid detergent fiber; WSC, water-soluble carbohydrate.

**Table 2:** Diet composition for Kazakh sheep.

Items	Content (%)
Ingredients	
Corn stalk	45.20
Alfalfa powder	13.00
Maize	17.20
Soybean meal	10.00
Cottonseed meal	6.00
CaHPO_4_	1.50
NaCl	0.50
Premix ^1^	1.00
Nutritional levels (% DM)	
ME ^2^, MJ/kg	8.51
CP	14.90
Ca	0.92
P	0.54
NDF	48.21
ADF	26.61

ME, metabolic energy; CP, crude protein; NDF, neutral detergent fiber; ADF, acid detergent fiber. ^1^ Premix provided the following contents per kilogram of the diet: vitamin A 100000 IU, vitamin D 21000 IU, vitamin E 850 mg, Fe 300 mg, Zn 3160 mg, Mn 400 mg, Cu 320 mg, I 50 mg, Se 18 mg, Co 30 mg. ^2^ ME value was calculated, and the other parameters (CP, Ca, P, NDF, and ADF) were measured.

**Table 3:** Effect of corn steep liquor supplementation levels on nutritional composition of corncob (%DM).

Items	CON	L1	L2	L3	L4	SEM	*p*-Value
DM (%)	34.08 ^c^	35.93 ^b^	35.99 ^b^	36.84 ^a^	36.56 ^a^	0.209	<0.001
CP (DM%)	3.78 ^c^	5.79 ^b^	6.17 ^b^	7.25 ^a^	7.83 ^a^	0.299	<0.001
WSC (DM%)	4.01 ^a^	2.77 ^b^	2.65 ^b^	2.83 ^b^	2.91 ^b^	0.136	<0.002
EE (DM%)	10.16 ^a^	9.73 ^ab^	8.89 ^bc^	8.32 ^c^	8.42 ^c^	0.222	<0.016
Ash (DM%)	2.92 ^d^	4.39 ^c^	4.82 ^b^	5.38 ^a^	5.25 ^a^	0.188	<0.001
NDF (DM%)	74.99 ^a^	68.73 ^b^	66.14 ^c^	63.00 ^d^	62.79 ^d^	0.936	<0.001
ADF (DM%)	37.23 ^a^	33.53 ^b^	32.28 ^c^	30.57 ^d^	30.41 ^d^	0.522	<0.001

Note: CON represents the control group; L1 denotes the group with 5% CSL added; L2, L3, and L4 correspond to the groups supplemented with 10%, 15%, and 20% CSL; ^a,b,c,d^ Values with different lowercase letters in the same row differ significantly (*p* < 0.05). DM, dry matter; CP, crude protein; WSC, water-soluble carbohydrate; EE, crude ether extract; Ash, crude ash; NDF, neutral detergent fiber; ADF, acid detergent fiber.

**Table 4:** Effect of corn steep liquor supplementation levels on fermentation parameters of corncob silage.

Items	CON	L1	L2	L3	L4	SEM	*p*-Value
pH	4.48 ^a^	4.31 ^b^	4.19 ^c^	4.16 ^c^	4.09 ^c^	0.032	<0.001
NH_3_-N (%TN)	2.84 ^a^	2.50 ^ab^	2.14 ^bc^	1.65 ^cd^	1.44 ^d^	0.131	<0.001
LA (mmol·mL^−1^)	2.05 ^b^	2.36 ^b^	3.82 ^a^	3.96 ^a^	4.87 ^a^	0.256	<0.001
AA (µg·mL^−1^)	14.98	13.64	19.80	22.10	13.71	1.274	0.104
PA (µg·mL^−1^)	3.21 ^ab^	4.21 ^ab^	5.04 ^a^	3.48 ^ab^	2.40 ^b^	0.300	<0.047
BA (µg·mL^−1^)	ND	ND	ND	ND	ND	-	-
LA/AA	0.14 ^b^	0.22 ^b^	0.20 ^b^	0.19 ^b^	0.40 ^a^	0.025	<0.004
AA/PA	5.73	3.11	3.94	8.78	6.49	0.912	0.323

Note: CON represents the control group; L1 denotes the group with 5% CSL added; L2, L3, and L4 correspond to the groups supplemented with 10%, 15%, and 20% CSL; Values with different lowercase letters in the same row differ significantly (*p* < 0.05). ND indicates that it is not detected. NH_3_-N, ammonia nitrogen/total nitrogen; LA, lactic acid; AA, acetic acid; PA, propionic acid; BA, butyric acid; LA/AA, lactic acid/acetic acid; AA/PA, acetic acid/propionic acid.

**Table 5:** Effect of corn steep liquor supplementation levels on the microbial diversity of corncob silage.

Item	CON	L1	L2	L3	L4	SEM	*p*-Value
Chao1	486.50 ^b^	527.96 ^b^	507.22 ^b^	554.27 ^ab^	601.44 ^a^	12.31	<0.017
ACE	493.26 ^b^	521.76 ^b^	497.61 ^b^	572.45 ^ab^	626.09 ^a^	14.58	<0.006
Shannon	2.97 ^a^	2.79 ^a^	2.48 ^b^	2.39 ^b^	1.93 ^c^	0.084	<0.001
Simpson	0.12 ^d^	0.17 ^cd^	0.22 ^bc^	0.23 ^b^	0.36 ^a^	0.018	<0.001

Note: CON represents the control group; L1 denotes the group with 5% CSL added; L2, L3, and L4 correspond to the groups supplemented with 10%, 15%, and 20% CSL. Values with different lowercase letters in the same row differ significantly (*p* < 0.05).

**Table 6:** Effects of corn steep liquor supplementation levels on rumen dry matter degradation of corncob silage.

Items	CON	L1	L2	L3	L4	SEM	*p*-Value
Ruminal degradation rate (%)							
4 h	12.88 ^d^	17.44 ^bc^	16.95 ^c^	19.27 ^ab^	20.03 ^a^	0.708	<0.001
8 h	17.44 ^d^	22.49 ^c^	25.54 ^b^	27.08 ^b^	29.94 ^a^	1.160	<0.001
12 h	37.57 ^a^	41.22 ^b^	36.66 ^a^	37.16 ^a^	37.56 ^a^	0.479	<0.001
24 h	47.68 ^a^	54.39 ^b^	55.54 ^ab^	53.43 ^b^	57.25 ^a^	0.918	<0.001
36 h	58.37 ^c^	69.92 ^a^	67.18 ^b^	68.08 ^b^	69.62 ^a^	1.156	<0.001
48 h	62.22 ^c^	74.13 ^a^	74.18 ^a^	71.61 ^b^	72.67 ^ab^	1.210	<0.001
Ruminal degradation parameters							
a (%)	3.52 ^c^	4.12 ^b^	3.76 ^bc^	7.23 ^a^	6.78 ^a^	0.429	<0.001
b (%)	69.44 ^c^	81.12 ^a^	81.38 ^a^	76.74 ^b^	75.44 ^b^	1.189	<0.001
c (%/h)	0.063 ^a^	0.045 ^bc^	0.044 ^bc^	0.038 ^c^	0.053 ^ab^	0.003	<0.007
ED (%)	49.10 ^b^	49.57 ^b^	49.35 ^b^	50.22 ^b^	53.12 ^a^	0.462	<0.007

Note: CON represents the control group; L1 denotes the group with 5% CSL added; L2, L3, and L4 correspond to the groups supplemented with 10%, 15%, and 20% CSL. a, the rapidly degraded part; b, the slow degradation part; c, the degradation rate of part b; ED, effective degradation rate. Values with different lowercase letters in the same row differ significantly (*p* < 0.05).

**Table 7:** Effects of corn steep liquor supplementation levels on ruminal crude protein degradation of corncob silage.

Items	CON	L1	L2	L3	L4	SEM	*p*-Value
Ruminal degradation rate (%)							
4 h	14.66 ^d^	25.19 ^b^	25.65 ^b^	21.83 ^c^	28.00 ^a^	1.252	<0.001
8 h	25.07 ^d^	29.99 ^c^	30.47 ^c^	38.90 ^a^	33.61 ^b^	1.227	<0.001
12 h	31.79 ^c^	43.76 ^a^	43.36 ^a^	42.24 ^b^	43.57 ^a^	1.236	<0.001
24 h	37.23 ^e^	52.69 ^d^	55.51 ^c^	58.35 ^b^	65.78 ^a^	2.518	<0.001
36 h	46.40 ^d^	63.94 ^c^	64.67 ^c^	66.25 ^b^	68.33 ^a^	2.118	<0.001
48 h	56.31 ^c^	71.17 ^b^	72.07 ^b^	74.69 ^a^	71.34 ^b^	1.758	<0.001
Ruminal degradation parameters							
a (%)	11.61 ^cd^	16.66 ^a^	15.41 ^b^	10.77 ^d^	12.18 ^c^	0.628	<0.001
b (%)	60.80 ^d^	67.10 ^a^	66.15 ^ab^	65.50 ^b^	63.60 ^c^	0.615	<0.001
c (%/h)	0.035 ^bc^	0.031 ^c^	0.040 ^b^	0.063 ^a^	0.070 ^a^	0.004	<0.001
ED (%)	41.22 ^d^	49.17 ^c^	52.27 ^b^	53.37 ^a^	54.11 ^a^	1.260	<0.001

Note: CON represents the control group; L1 denotes the group with 5% CSL added; L2, L3, and L4 correspond to the groups supplemented with 10%, 15%, and 20% CSL. a, the rapidly degraded part; b, the slow degradation part; c, the degradation rate of part b; ED, effective degradation rate. Values with different lowercase letters in the same row differ significantly (*p* < 0.05).

**Table 8:** Effects of corn steep liquor supplementation levels on ruminal neutral detergent fiber degradation of corncob silage.

Items	CON	L1	L2	L3	L4	SEM	*p*-Value
Ruminal degradation rate (%)							
4 h	7.37 ^b^	15.53 ^a^	13.71 ^a^	14.82 ^a^	15.01 ^a^	0.865	<0.001
8 h	13.67 ^c^	19.37 ^b^	17.69 ^b^	18.65 ^b^	21.80 ^a^	0.762	<0.001
12 h	28.25 ^ab^	25.06 ^bc^	21.09 ^c^	27.06 ^ab^	30.68 ^a^	1.006	<0.006
24 h	32.36 ^c^	38.30 ^b^	39.63 ^b^	37.02 ^b^	42.91 ^a^	0.982	<0.001
36 h	36.67 ^e^	47.78 ^c^	50.70 ^b^	44.18 ^d^	56.36 ^a^	1.763	<0.001
48 h	45.80 ^c^	55.32 ^b^	54.12 ^b^	55.40 ^b^	62.85 ^a^	1.476	<0.001
Ruminal degradation parameters							
a (%)	3.26 ^c^	8.80 ^a^	4.42 ^c^	9.93 ^a^	6.94 ^b^	0.706	<0.001
b (%)	47.91 ^b^	73.23 ^a^	69.92 ^a^	75.93 ^a^	74.32 ^a^	2.853	<0.001
c (%/h)	0.069 ^a^	0.029 ^b^	0.039 ^b^	0.029 ^b^	0.032 ^b^	0.004	<0.001
ED (%)	36.33 ^d^	37.29 ^cd^	38.37 ^c^	40.76 ^b^	43.25 ^a^	0.693	<0.001

Note: CON represents the control group; L1 denotes the group with 5% CSL added; L2, L3, and L4 correspond to the groups supplemented with 10%, 15%, and 20% CSL. a, the rapidly degraded part; b, the slow degradation part; c, the degradation rate of part b; ED, effective degradation rate. Values with different lowercase letters in the same row differ significantly (*p* < 0.05).

**Table 9:** Effects of corn steep liquor supplementation levels on the degradation of ruminal acid detergent fiber of corncob silage.

Items	CON	L1	L2	L3	L4	SEM	*p*-Value
Ruminal degradation rate (%)							
4 h	5.57 ^c^	9.22 ^b^	12.01 ^a^	12.03 ^a^	11.87 ^a^	0.686	<0.001
8 h	15.04 ^c^	14.97 ^c^	18.70 ^ab^	17.29 ^b^	19.44 ^a^	0.543	<0.001
12 h	19.76 ^c^	22.62 ^b^	24.09 ^b^	26.82 ^a^	27.35 ^a^	0.771	0.134
24 h	29.78 ^d^	34.96 ^c^	37.21 ^b^	39.76 ^a^	38.84 ^a^	0.969	<0.001
36 h	43.63 ^b^	45.19 ^ab^	47.80 ^a^	42.74 ^b^	46.53 ^a^	0.581	<0.008
48 h	49.23 ^c^	53.68 ^b^	53.42 ^b^	56.96 ^a^	57.66 ^a^	0.873	<0.001
Ruminal degradation parameters							
a (%)	1.58 ^b^	2.43 ^b^	4.84 ^a^	5.55 ^a^	5.97 ^a^	0.488	<0.001
b (%)	65.76 ^bc^	72.75 ^a^	64.17 ^c^	66.88 ^bc^	69.09 ^b^	0.895	<0.002
c (%/h)	0.031	0.032	0.038	0.039	0.039	0.001	0.170
ED (%)	34.15 ^c^	37.98 ^ab^	36.19 ^bc^	38.56 ^ab^	39.73 ^a^	0.650	<0.024

Note: CON represents the control group; L1 denotes the group with 5% CSL added; L2, L3, and L4 correspond to the groups supplemented with 10%, 15%, and 20% CSL. a, the rapidly degraded part; b, the slow degradation part; c, the degradation rate of part b; ED, effective degradation rate. Values with different lowercase letters in the same row differ significantly (*p* < 0.05).

## Data Availability

The sequence data reported in this study have been deposited in the Sequence Read Archive (SRA) under the accession number PRJNA1269722. The authors declare that no Gen AI was used in the creation of this manuscript.
